# Adverse effects of dasatinib on glucose-lipid metabolism in patients with chronic myeloid leukaemia in the chronic phase

**DOI:** 10.1038/s41598-019-54033-0

**Published:** 2019-11-26

**Authors:** Lu Yu, Jing Liu, Xiaojun Huang, Qian Jiang

**Affiliations:** 1 Peking University People’s Hospital, Peking University Institute of Hematology, National Clinical Research Center for Hematologic Disease, Beijing, China; 2 Department of Cardiology, Peking University People’s Hospital, Beijing, China

**Keywords:** Chronic myeloid leukaemia, Outcomes research

## Abstract

To explore the differences in glucose-lipid metabolism profiles among the 3 TKIs, we designed a retrospective study to compare the onset of hyperglycaemia, hypertriglyceridemia, hypercholesterolemia and hyper-low density lipoprotein (LDL)-cholesterolemia in the patients with normal baseline glucose-lipid profiles and had no medical record of cardio- or cerebro-vascular diseases and/or metabolic syndrome diseases, and identify variables associated with them. 370 chronic myeloid leukaemia patients receiving dasatinib, nilotinib or imatinib therapy ≥3 months were retrospectively reviewed. During TKI-therapy, the mean fasting glucose, triglyceride, cholesterol, and LDL-cholesterol levels increased significantly in both dasatinib and nilotinib cohorts compared with the imatinib cohort. In multivariate analyses, dasatinib was the factor significantly associated with both poor hyperglycaemia- and hypertriglyceridemia-free survival. In addition, nilotinib was significantly associated with more occurrences of hyperglycaemia and hypercholesterolemia; increasing age was significantly associated with more occurrences of hyperglycaemia and hypertriglyceridemia. We concluded that dasatinib, similar to nilotinib, has the adverse impact on glucose-lipid metabolism compared with imatinib.

## Introduction

Tyrosine kinase inhibitors (TKIs) have transformed the treatment of chronic myeloid leukaemia (CML). Patients with CML in chronic phase (CP) now have a similar life-expectancy to the general population and the number of people living with CML is increasing^1–5^. Despite an overall favourable safety profile of TKIs, adverse events (AEs) are common in TKI-therapy which may occur at the early phase or several years after the start of TKI treatment. There is increasing interest in the AEs of TKIs, especially in fatal toxicities on the second- or third- generation TKI-therapy compared to imatinib-therapy^6^. Compared with dasatinib and imatinib, nilotinib- and ponatinib-related cardio-cerebrovascular or peripheral artery events and abnormal glucose-lipid metabolism, which were significantly associated with cardiovascular events and metabolic syndrome, have been reported in many studies^7–12^. However, there is limited data on dasatinib, especially for Asian patients in whom CML was diagnosed at a relatively younger age compared with western people^13–21^. Differences in age, ethnicity, comorbidities, diet, genetics, drug used and treatment duration, may have important impacts on safety profile of TKI regarding glucose-lipid metabolism, which may be related to or as an early predictor of cardio-cerebrovascular events in the future. Currently, several TKIs have been approved as the first-line therapy in patients with CML in many countries; while imatinib and nilotinib, first-line option; and dasatinib, second- or third-line option in China. To explore the differences on glucose-lipid metabolism profiles among the 3 TKIs, we designed a retrospective study to compare the onset of hyperglycaemia, hypertriglyceridemia, hypercholesterolemia and hyper-low density lipoprotein (LDL)-cholesterolemia in the patients with normal baseline glucose-lipid profiles during TKI-therapy and with no medical record of cardio- or cerebro-vascular diseases and/or metabolic syndrome diseases, and identify variables associated with them.

## Materials and Methods

### Patients and methods

Data of patients with CML in the chronic phase receiving imatinib (Glivec^@^, Novartis), nilotinib (Tasigna^@^, Novartis) or dasatinib (Sprycel^@^, Bristol-Myers Squibb) as first-, second- or third-line TKI-therapy ≥3 months from May 2001 to August 2017 at Peking University People’s Hospital were retrospectively reviewed. Patients with normal glucose-lipid values at baseline and no medical history record of cardio- or cerebro-vascular diseases and/or metabolic syndrome, and laboratory test followed regularly were included in this study. Hematologic, cytogenetic and molecular responses were monitored according to European LeukemiaNet recommendations for the management of chronic myeloid leukemia^22^. Dosages of TKIs were adjusted according to patients’ response and intolerance (excluding abnormal glucose-lipid metabolism). Laboratory examinations were collected, including levels of glucose, triglyceride, cholesterol and LDL-C in blood, under fasting conditions before TKI-therapy, every 1–2 weeks for the first 3 months after starting TKI-therapy and every 3–6 months thereafter until the patient changed to other therapy. Demographics (sex, age), CML data (diagnosis and disease phase), comorbidities, therapy(ies) history, TKI-therapy (interval from diagnosis to starting, drug, treatment duration, and dosage change) were also collected and analysed. In this study, we focused on the metabolism function change during TKI-therapy. All patients signed informed consent forms before TKI-therapy. The study protocol was approved by the Ethics Committee of Peking University People’s Hospital.

### Definitions

According to the upper limits of normal (ULN) in the lab of Peking University People’s Hospital, hyperglycaemia, hypertriglyceridemia, hypercholesterolemia and hyper-LDL-cholesterolemia were defined as the laboratory test values of fasting glucose (GLU), triglyceride (TG), cholesterol (CHO), and LDL-cholesterol (LDL-C) higher than the ULN presented on two or more occasions at least 3 months apart, respectively. The normal range of GLU, TG, CHO and LDL-C were 3.3–6.1 mmol/L, 0.45–1.7 mmol/L, 2.9–6.2 mmol/L, and 1.9–4.1 mmol/L, respectively. The severity of hyperglycaemia, hypertriglyceridemia and hypercholesterolemia was assessed according to Common Terminology Criteria for Adverse Events (CTCAE) 4.0. As these AEs may occur over time on TKI-therapy, hyperglycaemia-, hypertriglyceridemia-, hypercholesterolemia-, and hyper-LDL-cholesterolemia-free survivals were defined as the time between TKI-therapy initiation and the onset of the AEs for analysing the glucose-lipid metabolic abnormality during TKI-therapy.

### Statistical analyses

Descriptive analysis results are presented as median (range) or number (percent) as appropriate. Pearson Chi-squared (for categorical variables) and Mann-Whitney U/Kruskal-Wallis tests (for continuous variables) were used to measure between-group differences. Statistically significant differences in the levels of GLU, TG, LDL-C, and CHO were calculated with a paired-samples Student’s T test between the values at baseline and at each time point in each cohort. The log-rank test was used to assess statistical significance in the time-to-event analyses. Univariate analyses including TKI used, sex, age, interval from diagnosis to starting TKI within 6 months or not, interval from diagnosis to taking this TKI, TKI used as first-line versus second- or third- line therapy and TKI-dosage change were performed to determine variables associated with the onset of hyperglycaemia, hypertriglyceridemia, hypercholesterolemia and hyper-LDL-cholesterolemia. Variables associated at a level of *p* < 0.2 were included in the Cox regression model to identify variables significantly associated with the abnormalities of glucose-lipid metabolism profiles. Factors with an effect significant at the *p* < 0.05 were interpreted as independently predicting outcomes. All analyses were conducted with SPSS version 22.0 software (SPSS Inc., Chicago, IL, USA).

### Informed consent

Informed consent was obtained from all individual participants included in the study.

### Ethical approval

All procedures performed in studies involving human participants were in accordance with the ethical standards of the institutional and/or national research committee and with the 1964 Helsinki declaration and its later amendments or comparable ethical standards.

## Results

### Patient characteristics

A total of 370 CML-CP patients receiving imatinib (n = 225, 61%), dasatinib (n = 43, 12%) or nilotinib (n = 102, 27%) as front- (total, n = 296, 80.0%; including imatinib, n = 225; dasatinib, n = 2; and nilotinib, n = 69), second-line (total, n = 58, 15.7%; including dasatinib, n = 29 and nilotinib, n = 29) or third-line (total, n = 16, 4.3%; including dasatinib, n = 12 and nilotinib, n = 4) therapy with normal glucose-lipid values at baseline and no medical history record of cardio-cerebrovascular diseases and/or metabolic syndrome were included in this study. As the second-line TKI therapy, imatinib and then dasatinib, n = 28; nilotinib and then dastinib, n = 1; imatinib and then nilotinib, n = 29. As the third-line TKI therapy, imatinib, nilotinib and then dasatinib, n = 12; imatinib, dasatinib and then nilotinib, n = 4. Those receiving current TKI of dasatinb or nilotinib as second- ot third-line TKI therapy had normal glucose-lipid values during previous TKI therapy. The median age was 36 years (range, 18–80 years), and 239 (65%) patients were male. There were some differences on characteristics among the 3 cohorts, including more patients receiving dasatinib as second- or third-line TKI-therapy (*p* = 0.001), the longest interval from diagnosis to starting current TKI-therapy in the dasatinib cohort (*p* < 0.001), the most common TKI-dose reduction in the nilotinib cohort due to non-glucose-lipid toxicities (*p* < 0.001), and the shorter follow-up period in the dasatinib (median, 24 months; range, 3–114 months) and nilotinib (median, 21months; range, 3–90 months) cohorts compared with the imatinib cohort (median, 48 months; range, 3–188 months) (*p* < 0.001) (Table 1).

**Table 1:** Patients’ characteristics.

Variables	Dasatinib, N (%)	Nilotinib, N (%)	Imatinib, N (%)	*P value*
Patient number	43 (11.7)	102 (27.6)	225 (60.8)	
First-line therapy	2 (4.7)	69 (67.6)	225 (100)	<0.001
Second-line therapy*	29 (67.4)	29 (28.4)	0	
Third-line therapy**	12 (27.9)	4 (3.9)	0	
Male	29 (67.4)	66 (64.7)	144 (64.0)	0.910
Age, median (range), year	32 (18–68)	35 (18–74)	38 (18–80)	0.346
18–<30	14 (32.6)	30 (29.4)	70 (31.1)	
30–<40	17 (39.5)	30 (29.4)	57 (25.3)	
40–49	7 (16.3)	24 (23.5)	52 (23.1)	
50–59	2 (4.7)	14 (13.7)	30 (13.3)	
≥60	3 (7.0)	4 (3.9)	16 (7.1)	
Interval from diagnosis to starting TKI-therapy (months)				0.001
<6	30 (69.8)	89 (87.3)	204 (90.7)	
≥6	13 (30.2)	13 (12.7)	21 (9.3)	
Interval from diagnosis to current TKI-therapy (months)				<0.001
<6	11 (25.6)	62 (60.8)	204 (90.7)	
≥6	32 (74.4)	40 (39.2)	21 (9.3)	
Dosage reduction	3 (7.0)	25 (24.5)	21 (9.3)	<0.001
Therapy duration, median (range), months	24 (3–114)	21 (3–90)	48 (3–188)	<0.001

Abbreviation: TKI, tyrosine kinase inhibitor.

*As the second-line TKI therapy, imatinib and then dasatinib, n = 28; nilotinib and then dastinib, n = 1; imatinib and then nilotinib, n = 29.

**As the third-line TKI therapy, imatinib, nilotinib and then dasatinib, n = 12; imatinib, dasatinib and then nilotinib, n = 4.

### Glucose-lipid metabolic abnormalities

There was no significant difference on fasting GLU, TG, LDL-C, and CHO levels at baseline among the 3 cohorts. With the median follow-up periods of 24 months (range, 3–114 months) in the dasatinib cohort, 21 months (range, 3–90 months) in the nilotinib cohort, and 48 months (range, 3–188 months) in the imatinib cohort, respectively, the glucose-lipid metabolic abnormalities occurred in the 3 cohorts as follows:

### Hyperglycaemia

**Figure 1:**
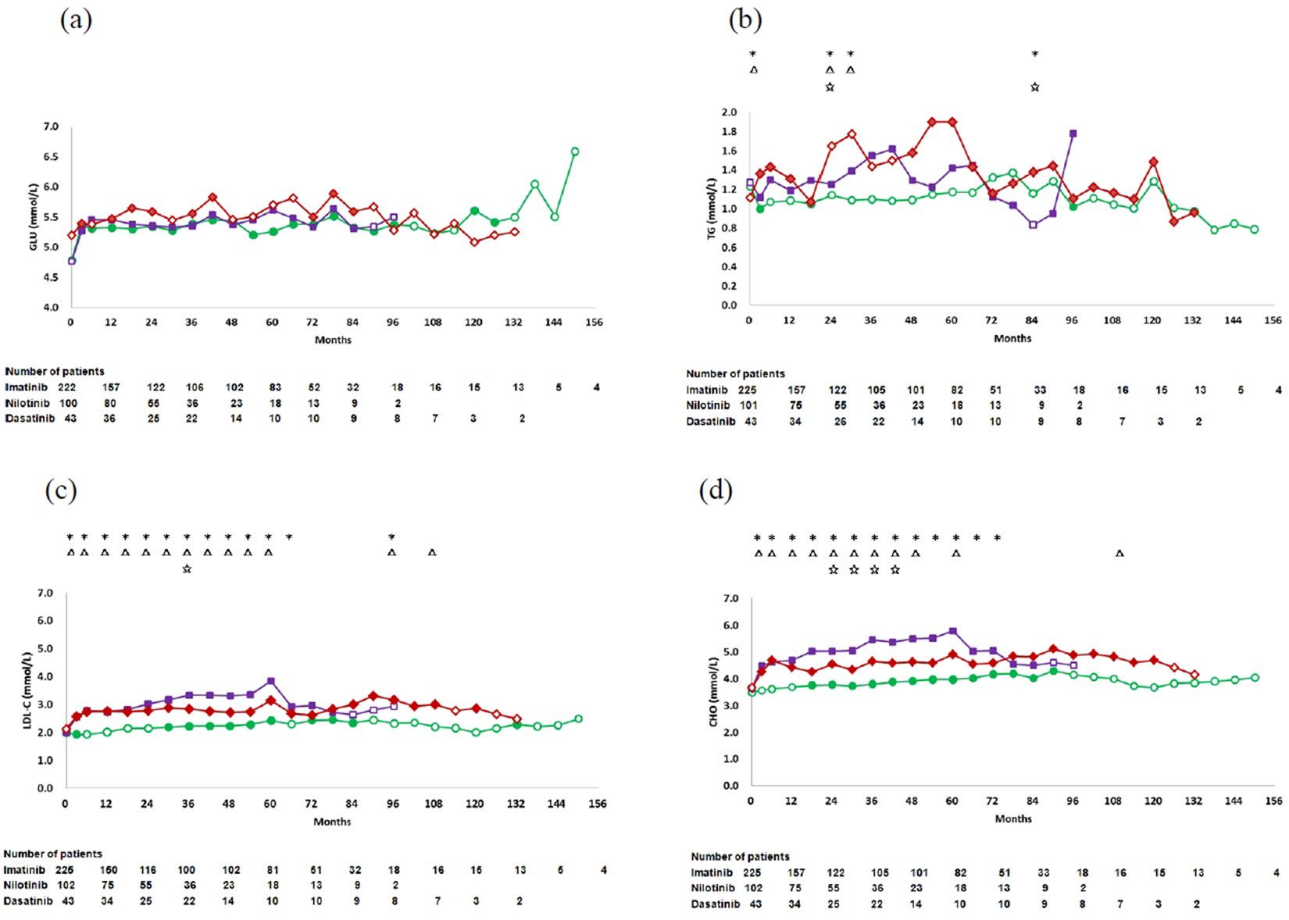
Changes in glucose (GLU) (**a**), triglyceride (TG) (**b**), low density lipoprotein cholesterol (LDL-C) (**c**) and cholesterol (CHO) (**d**) levels in the dasatinib, nilotinib and imatinib cohorts. Statistically significant differences were calculated with a paired-samples Student’s T test between the baseline and each time point, solid dot indicate *P* < 0.05.  imatinib,  nilotinib,  dasatinib. * statistically significant differences among the 3 cohorts (*P* < 0.05), △ statistically significant differences between the dasatinib and imatinib cohorts (*P* < 0.05), **☆** statistically significant differences between the dasatinib and nilotinib cohorts (*P* < 0.05).

**Figure 2:**
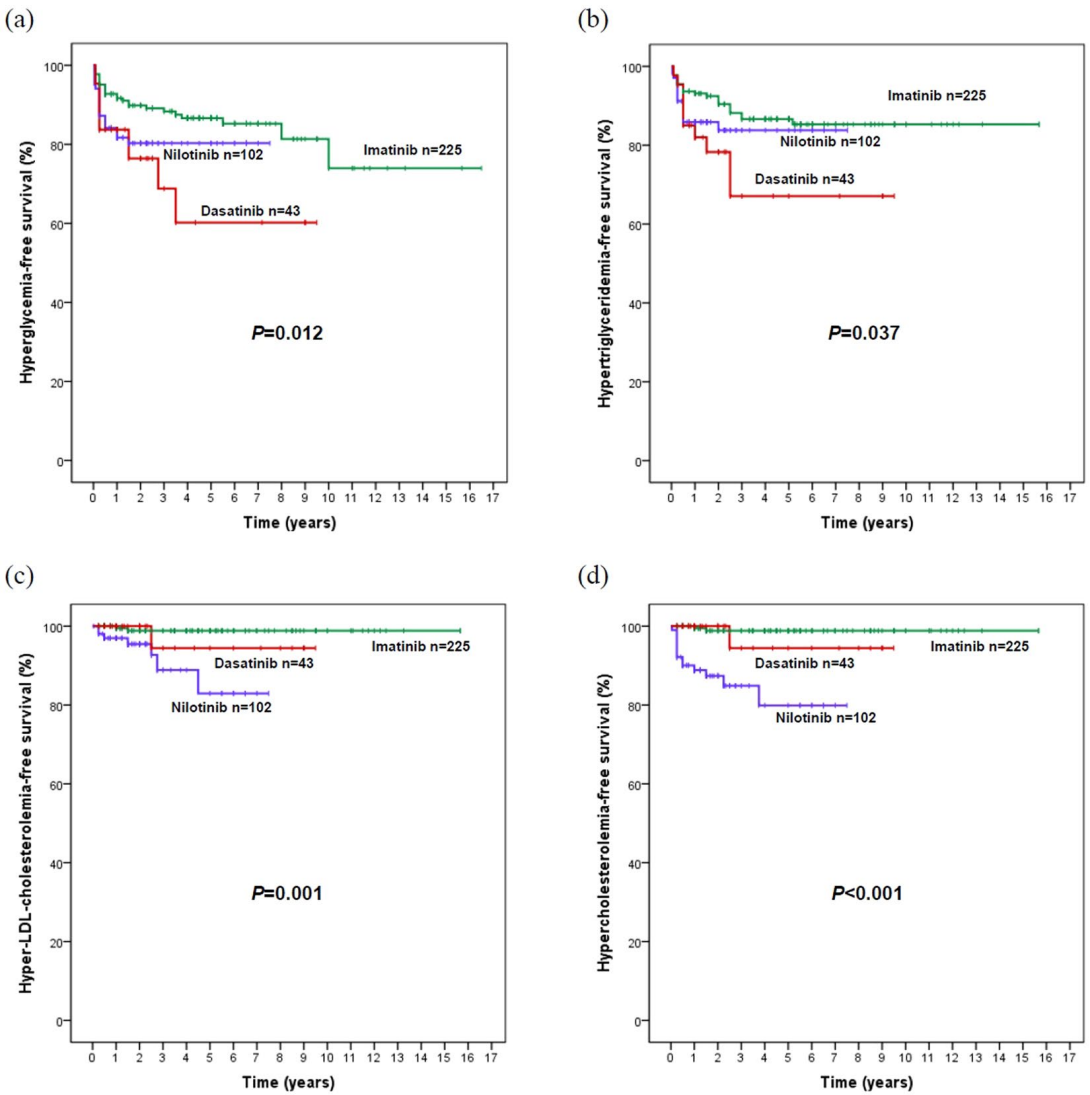
Hyperglycaemia- (**a**), hypertriglyceridemia- (**b**), hyper-low density lipoprotein (LDL)-cholesterolemia- (**c**), and hypercholesterolemia-free survival (**d**) by dasatinib, nilotinib and imatinib.

The mean glucose levels significantly increased in the 3 cohorts at most time points during TKI-therapy compared with the baseline. There was no significant difference at each time point among the 3 cohorts by Kruskal-Wallis tests (Fig. 1). 11 (25.6%) patients developed hyperglycaemia at a median of 3 months (range, 1–42 months) in the dasatinib cohort; 19 (18.6%), at 3 months (range, 0.5–18 months) in the nilotinib cohort; and 28 (12.4%), at 6 months (range, 0.5–120 months) in the imatinib cohort. According to CTCAE, none in the dasatinib cohort experienced ≥ grade 2 of hyperglycaemia; however, 3 patients experienced hyperglycaemia of grade 2 (n = 2) or grade 3 (n = 1) in the nilotinib cohort, and 1 patient (grade 2) in the imatinib cohort. The 3-year probability of hyperglycaemia-free survival in the dasatinib cohort (66% [95% CI: 46–86%]) was the lowest among the 3 cohorts (*p* = 0.012), although there was no statistical difference between the dasatinib and the nilotinib cohorts (76% [95% CI: 67–85%]) (*p* = 0.387), which were significantly lower than that in the imatinib cohort (88% [95% CI: 84–92%]) (Fig. 2). There was no significant difference on the 3-year probability of hyperglycaemia-free survival in dasatinib cohort (*p* = 0.362) and the nilotinib cohort (*p* = 0.405) by lines of TKI therapy, respectively. In a multivariate analysis, dasatinib (HR = 2.6, 95% CI: 1.3–5.2, *p* = 0.009), nilotinib (HR = 2.0, 95% CI: 1.1–3.7, *p* = 0.019) and increasing age (≥40 years, HR = 2.0–6.7, [0.9–16.7], *p* = < 0.001–0.07) were factors significantly associated with poor hyperglycaemia-free survival.

### Hypertriglyceridemia

The mean TG levels significantly increased in both dasatinib and nilotinib cohorts at most time points on therapy compared with the baseline. There were significant differences on the TG levels among the 3 cohorts at 3, 24, 30, and 84 months, including the TG levels in the dasatinib cohort were significantly higher than those in the nilotinib or imatinib cohort at several time points (Fig. 1). 10 (23.3%) patients developed hypertriglyceridemia at a median of 6 months (range, 1–30 months) in the dasatinib cohort; 15 (14.7%), at 3 months (range, 0.5–24 months) in the nilotinib cohort, and 25 (11.2%), at 6 months (range, 0.5–62 months) in the imatinib cohort. 5 patients experienced ≥ grade 2 of hypertriglyceridemia in the dasatinib cohort; 7, in the nilotinib cohort; and 8, in the imatinib cohort. The 3-year probability of hypertriglyceridemia-free survival in the dasatinib cohort (62% [95% CI: 42–82%]) was the lowest compared with those in the nilotinib cohort (82% [95% CI: 74–90%]) and imatinib cohort (87% [95% CI: 82–92%]) (*p* = 0.037). There was no difference between the nilotinib and imatinib cohorts (*p* = 0.270) (Fig. 2). There was no significant difference on the 3-year probability of hypertriglyceridemia-free survival in dasatinib cohort (p = 0.993) and the nilotinib cohort (p = 0.354) by lines of TKI therapy, respectively. In a multivariate analysis, dasatinib (HR = 2.7, 95% CI: 1.3–5.7, *p* = 0.008) and increasing age (50–60 years, HR = 2.4, 95% CI: 1.0–5.5, *p* = 0.041) were factors significantly associated with poor hypertriglyceridemia-free survival.

### Hyper-LDL-cholesterolemia

The mean LDL-C level significantly increased in the 3 cohorts during the TKI-therapy compared with the baseline, especially in both dasatinib and nilotinib cohorts. The LDL-C levels in the dasatinib cohort were significantly higher than those in the imatinib cohort at 3–60, 96 and 108 months, while similar to those in the nilotinib cohort except at 36 months (Fig. 1). One (2.3%) patient developed hyper-LDL-cholesterolemia at 30 months in the dasatinib cohort; 7 (6.9%), at a median of 18 months (range, 3–54 months) in the nilotinib cohort; and 2 (0.9%), at 12 months and 18 months in the imatinib cohort, respectively. There was no significant difference on the 3-year probabilities of hyper-LDL-cholesterolemia-free survival in dasatinib cohort (*p* = 0.593) and the nilotinib cohort (*p* = 0.608) by lines of TKI therapy, respectively. Although there was a significant difference on the 3-year probabilities of hyper-LDL-cholesterolemia-free survival among the dasatinib cohort (94% [95% CI: 88–100%]), imatinib (99% [95% CI: 98–100%]) cohort, and the nilotinib cohort (88% [95% CI: 78–97%]) (*p* = 0.001) (Fig. 2), no variable was identified associated with the onset of hyper-LDL-cholesterolemia in the multivariate analysis.

### Hypercholesterolemia

The mean CHO levels significantly increased in the 3 cohorts during the TKI-therapy compared with the baseline, especially in both dasatinib and nilotinib cohorts. The CHO levels in the dasatinib cohort were significantly higher than those in the imatinib cohort at 3–48, 60 and 108 months, while lower than those in the nilotinib cohort at 24–42 months (Fig. 1). One (2.3%) patient developed hypercholesterolemia at 30 months in the dasatinib cohort; 4 (13.7%), at a median of 3 months (range, 0.5–45 months) in the nilotinib cohort; and 2 (0.9%), at 12 months and 18 months in the imatinib cohort, respectively. According to CTCAE, 3 patients experienced ≥grade 2 of hypercholesterolemia in the nilotinib cohort; however, none in the dasatinib or imatinib cohort. There was a significant difference on the 3-year probabilities of hypercholesterolemia-free survival in the dasatinib cohort (94% [95% CI: 88–100%]), imatinib cohort (99% [95% CI: 98–100%]) and nilotinib cohort (82% [95% CI: 72–92%]) (*p* < 0.001) (Fig. 2). There was no significant difference on the 3-year probabilities of hypercholesterolemia-free survival in dasatinib cohort (*p* = 0.533) and the nilotinib cohort (*p* = 0.527) respectively as second-line treatment versus as third-line treatment. In a multivariate analysis, nilotinib (HR = 19, 95% CI: 4.3–85.3, *p* < 0.001) was the only factor associated with poor hypercholesterolemia-free survival.

During the follow-up period, there was no cardio-cerebrovascular event occurred, and no concomitant medication was prescribed for the mild or intermediate glucose-lipid metabolic abnormalities in all patients.

## Discussion

We explored the effects of dasatinib on fasting blood GLU, TG, CHO and LDL-C in relatively young CML-CP patients with normal baseline and no cardio-vascular disease or metabolic syndrome, and compared with nilotinib and imatinib. We found that dasatinib was significantly associated with an increased onset of hyperglycaemia and hypertriglyceridemia compared with imatinib. In addition, nilotinib was significantly associated with more occurrences of hyperglycaemia and hypercholesterolemia; increasing age was significantly associated with more occurrences of hyperglycaemia and hypertriglyceridemia.

There are several studies about the adverse impacts of nilotinib on glucose-lipid metabolism^7,20,23,24^. However, data regarding glucose-lipid metabolic abnormalities focused on dasatinib is limited. Iurlo *et al*. reported in a study which included 107 patients (40 treated with dasatinib; 36, nilotinib; and 92, imatinib) at a median age of 56 years, although fasting plasma glucose, insulin, C-peptide, and Homeostasis Model Assessment-Insulin Resistance were significantly higher in the nilotinib group than those in the dasatinib and imatinib groups, LDL-C did not differ significantly between the dasatinib and nilotinib groups, which was higher than that in the imatinib group^20^. We confirmed TKI-related hyper-LDL-cholesterolemia by using more sensitive and accurate assessment methods of higher than the ULN presented on two or more occasions at least 3 months apart and event-free survival rate (refers to hyper-LDL-cholesterolemia-free survival), because drug-related glucose lipid abnormalities may develop over time. In our current study, dasatinib was also associated with a higher onset of hyperglycaemia and hypertriglyceridemia compared with imatinib. Because dasatinib and nilotinib share the same mechanism of targeting ABL and BCR-ABL, we speculate that dasatinib might have the similar effect and mechanism on IR and the IR metabolic pathway. It has been reported that nilotinib was associated with hyperinsulinaemia and insulin resistance, which likely occurred on the post-receptor level. A study including 10 CML patients without a medical history of diabetes mellitus clarified that the mechanism of impaired glucose metabolism and dyslipidaemia occurs via rapidly developed tissue insulin resistance and compensatory hyperinsulinaemia. Some studies *in vitro* indicate c-ABL was involved in the insulin receptor (IR) signalling pathway and enhances the IR metabolic pathway. However, the effect on the IR pathway of each TKI has not yet been well understudied^9^. Various animal models have been used to evaluate what role tyrosine kinases play in the regulation of glucose-lipid levels. Krishnamurthy *et al*. illustrated with *in vitro* studies that c-kit tyrosine kinase was essential for β-cell survival in the pancreas. A mouse with a c-kit point mutation that diminishes the receptor’s kinase activity exhibited glucose intolerance, impaired insulin secretion and a reduction in β-cell mass^25^. However, the real, accurate mechanism has not been found in human studies.

Some studies reported that dasatinib can decrease blood glucose levels^19,26,27^. Keiko *et al*. reported a rapid amelioration of hyperglycaemia facilitated by dasatinib in a CML patient with diabetes mellitus^27^. However, in our current study dasatinib was associated with a higher onset of hyperglycaemia and hypertriglyceridemia. This finding may be due to the different population regarding age, comorbidities, ethnic and genetics which are associated with different risk or susceptibility to glucose-lipid dysfunction or prediabetes, and the assessing method of event-free survival used in our study. All patients in our study had a normal glucose-lipid baseline and no medical record of diabetes. The mechanism might be different between the patients with or without the comorbidity of diabetes.

Even in these younger patients with no medical record of pre-existing metabolic syndrome and a short follow-up period, abnormal glucose-lipid metabolism occurred in the dasatinib and nilotinib cohorts. Therefore, for those receiving dasatinib or nilotinib therapy in the pursuit of treatment-free remission, the risk of metabolic syndrome or cardio-cerebrovascular events should be taken into consideration, and laboratory index of glucose-lipid metabolism should be closely monitored.

Our study has some limitations. First, there were some differences in baseline characteristics among the 3 cohorts in this retrospective study. Second, there were relatively small patient numbers in the dasatinib and nilotinib cohorts. Third, patients with no medical history record of cardio- or cerebro-vascular diseases and/or metabolic syndrome were included in this study; however, some patients in very early stage of these diseases could not be excluded. Fourth, several other factors which had not been collected may contribute to modifications of glucose and blood lipids equilibrium, such as persistent diet or physical activity change, personal predisposition, and a gain of weight.

We concluded that dasatinib, similar to nilotinib, has adverse impact on glucose-lipid metabolism when compared with imatinib. These data favour using imatinib over dasatinib and nilotinib in older patients, even in younger CML patients with normal baseline glucose-lipid levels and without cardio- or cerebro-vascular diseases and/or metabolic syndrome who want to pursue TFR, laboratory index of glucose-lipid metabolism should be monitored closely.
